# Oral Infection, Oral Pathology and Salivary Diagnostics of Mpox Disease: Relevance in Dentistry and OMICs Perspectives

**DOI:** 10.3390/ijms241814362

**Published:** 2023-09-21

**Authors:** Marcelo Augusto Garcia-Junior, Bruno Silva Andrade, Marco Guevara-Vega, Igor Santana de Melo, Thúlio M. Cunha, Ana Carolina Gomes Jardim, Robinson Sabino-Silva

**Affiliations:** 1 Innovation Center in Salivary Diagnostics and Nanobiotechnology, Laboratory of Nanobiotechnology – “Luiz Ricardo Goulart”, Department of Physiology, Institute of Biomedical Sciences, Federal University of Uberlandia, Uberlândia 38496-017, Brazilmarco.guevara.vega@gmail.com (M.G.-V.); 2 Laboratory of Bioinformatics and Computational Chemistry, Department of Biological Sciences, State University of Southwest of Bahia (UESB), Jequié 45083-900, Brazil; 3 Department of Histology and Embryology, Institute of Biological Sciences and Health, Federal University of Alagoas (UFAL), Maceió 57072-260, Brazil; 4 Department of Pulmonology, School of Medicine, Federal University of Uberlandia, Uberlândia 38496-017, Brazil; 5 Laboratory of Antiviral Research, Department of Microbiology, Institute of Biomedical Sciences, Federal University of Uberlandia, Uberlândia 38496-017, Brazil

**Keywords:** monkeypox, saliva, oral pathology, multiomics, proteomics, dentistry, virology

## Abstract

In this narrative review, we aim to point out the close relationship between mpox virus (MPXV) infection and the role of saliva as a diagnostic tool for mpox, considering the current molecular approach and in the perspective of OMICs application. The MPXV uses the host cell’s rough endoplasmic reticulum, ribosomes, and cytoplasmic proteins to replicate its genome and synthesize virions for cellular exit. The presence of oral mucosa lesions associated with mpox infection is one of the first signs of infection; however, current diagnostic tools find it difficult to detect the virus before the rashes begin. MPXV transmission occurs through direct contact with an infected lesion and infected body fluids, including saliva, presenting a potential use of this fluid for diagnostic purposes. Currently available diagnostic tests for MPXV detection are performed either by real-time quantitative PCR (RT-qPCR) or ELISA, which presents several limitations since they are invasive tests. Despite current clinical trials with restricted sample size, MPXV DNA was detected in saliva with a sensitivity of 85%–100%. In this context, the application of transcriptomics, metabolomics, lipidomics, or proteomics analyses coupled with saliva can identify novel disease biomarkers. Thus, it is important to note that the identification and quantification of salivary DNA, RNA, lipid, protein, and metabolite can provide novel non-invasive biomarkers through the use of OMICs platforms aiding in the early detection and diagnosis of MPXV infection. Untargeted mass spectrometry (MS)-based proteomics reveals that some proteins also expressed in saliva were detected with greater expression differences in blood plasma when comparing mpox patients and healthy subjects, suggesting a promising alternative to be applied in screening or diagnostic platforms for mpox salivary diagnostics coupled to OMICs.

## 1. Introduction

The mpox disease was first detected in cynomolgus monkeys in 1958 and exhibited skin lesions as a typical clinical feature. Due to the pioneering discovery in monkeys, the term “monkeypox virus” was coined in 1958, and it was later renamed as “mpox” by the World Health Organization (WHO) in November 2022 and included in the ICD-10 [1,2]. The first reported mpox disease case consisted of an infant from the Democratic Republic of the Congo in 1970, and since then, there have been several other mpox virus (MPXV) outbreaks, frequently limited to Africa, but only recently became a global threat [3,4].

From a historical perspective, from 1970 to 2000, less than 1000 cases were confirmed, mostly in endemic regions caused by mpox viral clade I, without detection in other continents. Subsequently, the number of confirmed mpox cases increased to over 18,000 between 2000 and 2019 with predominant detection in Africa and confirmed cases in North America, Europe, and Asia [2,5,6]. The first human detection of mpox outside the African endemic regions was described in the United States in 2003 [7]. In this mpox outbreak, there was an outbreak recording 47 cases in the United States that were traced back to prairie dogs kept in contact with exotic rodents imported from Ghana [6]. Until November 2021, reports of human-to-human transmission of mpox were still incipient, except for a cluster of cases in the United Kingdom, when a family member was infected during a trip to Nigeria and transmitted the virus to other two relatives [8].

In the 2022/2023 simultaneous multi-country outbreaks of mpox over 88,000 cases and 149 deaths have been reported in more than 110 countries worldwide until July 2023 [9,10,11]. In this context, the United States has been the country with the highest number of cases reporting more than 30,000 cases, followed by Brazil and Spain with about 26,000 and 7000 cases, respectively [11]. On 23 July 2022, the worldwide mpox epidemic was designated a Public Health Emergency of International Concern (PHEIC) by the WHO. However, on 11 May 2023, the Emergency Committee on mpox reported that the outbreak is no longer a PHEIC because of a consistent drop in cases. A strategy plan and technical guide materials for mpox were prepared by the WHO, pointing to surveillance, diagnostics, risk communication, and community involvement as crucial for preventing recurrence. Although longer-term control and eradication are difficult, continued efforts lower the likelihood of a worldwide mpox comeback [11]. According to WHO, there are two different circulating clades of the virus causing current infections: clade I, associated with lower transmission; and clade II, related to higher spreading and most of the worldwide spread of the virus [9,10,12]. Altogether with the decrease in smallpox vaccination, the current MPXV outbreak is potentially related to the action of host apolipoprotein B mRNA-editing catalytic polypeptide-like 3 (APOBEC3) enzymes in the viral genome [13]. The unprecedented 2022/2023 simultaneous multi-country outbreaks of mpox registered a high level of human-to-human transmission, which was related to a high index of case fatality rates and organ damage issues [2]. Now, due to the 2022/2023 mpox outbreak, most of the cases do not present a relation to traveling to endemic regions, and human-to-human transmission is mostly present in lesions on the tongue, oral, and perioral regions, despite sexual transmission and HIV co-infection risk factors are still in need of elucidation [14,15].

Within this context, this narrative review aims to approach MPXV structure and replication alongside insights into oral transmission and symptoms, placing saliva at the core of diagnostic perspectives. Additionally, we describe the current methods of mpox diagnostic using molecular tools and present the perspective of Omics application in mpox salivary diagnostics and screening.

## 2. Study Design and Search Strategy

We conducted a narrative review of the literature following the PRISMA (Preferred Reporting Items for Systematic Review and Meta-analyses) guidelines when applicable. The aim of our review was to assess research papers—including various types of studies—that would enlighten the relationship between MPXV and saliva in order to better understand the potential use of saliva coupled with OMICs as a means of mpox diagnosis. We also included articles related to MPXV structure and replication, oral transmission, and systemic and oral symptoms.

From October 2022 to August 2023, the search for review was performed with the following keywords: “monkeypox” and/or “mpox”, “saliva”, “multiomics”, “proteomics”, “diagnostics” and “dentistry” into PubMed, MEDLINE, and Google Scholar databases. Further to original research articles of different types of studies, review articles, and national (UK and US) or international (WHO) guidelines were consulted, as well as gray literature. MeSH terms were chosen as the general controlled vocabulary and keywords, while language, year, and publication type restrictions were not applied to the searches.

## 3. Viral Structure, MPXV Entry into Host Cells, and MPXV Replicative Cycle

MPXV belongs to the Orthopoxvirus genus and Poxviridae family, characterized by having an oval-shaped structure and double-stranded DNA with 200 genes packed within 200,000 base pairs [6]. Specifically, this large genome is composed of a linear double-stranded DNA coupled with a hairpin on both 5′ and 3′ ends by a covalent interaction with inverted tandem repeats (ITS). It was suggested that MPXV is the largest virus capable of infecting human cells with a significant tissue tropism. As expected, several mammalian cell lines permit a viral entry [16]. Considering the two existent clades of MPXV, although infection and mortality rates are different, their genomic sequence is only 0.5% different in regions that have important virulent genes [17,18,19]. The WHO indicates that the global 2022/2023 mpox outbreak was caused by MPXV clade IIb. MPXV also contains two distinct viral particles: the mature virion (MV) and the enveloped virion (EV), and either of them uses different mechanisms to enter the host cells [20]. While the MV contains more than 20 viral proteins on the surface of its single lipid membrane, EV contains the MV structures plus a secondary outer covering membrane presenting 6 viral proteins. The intracellular mechanisms responsible for producing EV and MV present distinct viral protein compositions. In this context, although the majority of the MPXV virions are MVs, an effective immune defense response against mpox needs specific antibodies targeting both MV and EV [21,22].

About 50% of the genes in the genome of MPXV genome are responsible for its replication, and the remaining genes are related to interactions between the virus and the host cell [6,23]. The interaction between the surface components in the viral structure and the host cell, allowing the viral membrane to fuse with plasma or endosomal membrane in host cells, is required for viral replication. The surface proteins A26, A27, D8, and H3 can interact with glycosaminoglycans in host cells promoting cellular attachment [24,25]. Therefore, these surface proteins have already been considered targets for effective orthopoxvirus vaccines, including against MPXV [23].

**Figure 1:**
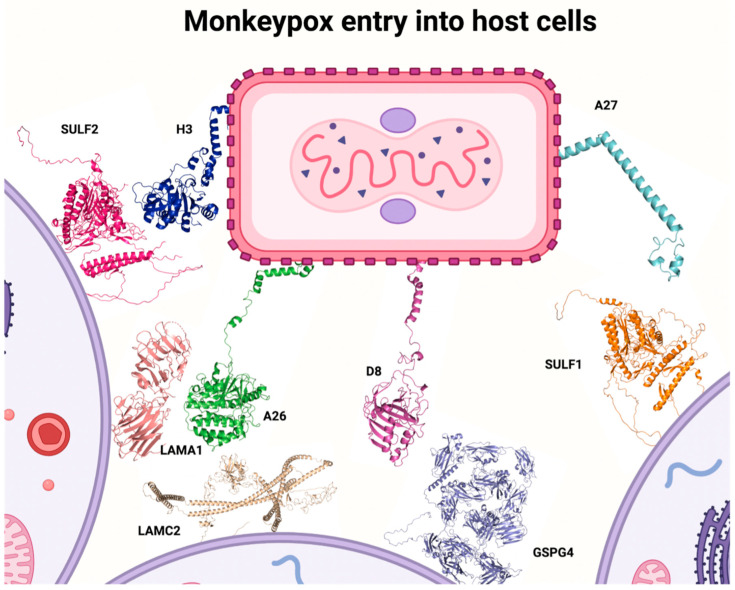
MPXV structure with surface proteins A26, A27, D8, and H3 and its respective interaction with glycosaminoglycans (created by the authors).

In order to understand the viral entry mechanisms for MPXV, it is essential to understand the interactions between glycosaminoglycans and viral surface proteins. In this context, A26 interacts with laminin on its alpha 1 and gamma 2 subunits—expressed in humans by *LAMA1* and *LAMC2* genes; A27 and H3 proteins interact with heparan sulfate, specifically on sulfatases 1 and 2—coded by *SULF1* and *SULF2* genes; D8 interacts with chondroitin sulfate on its proteoglycan 4 receptor—coded by *CSPG4* gene [23,25,26,27]. Considering MPXV viral replication mechanisms, Figure 1 illustrates MPXV structure with surface proteins A26, A27, D8, and H3 and its respective interaction with genes/proteins of glycosaminoglycans that were described in oral mucosa, salivary gland, and tongue. Since the MPXV mechanism of entry into cells is not completely elucidated, it is difficult to measure the importance of these glycosaminoglycans on specific regions of the human body, however, these genes and/or receptors are located in the oral mucosa, salivary glands, and tongue, which suggests that the oral cavity and related tissues could play an important role on MPXV infection (Table 1 and Table 2) [28,29].

After MPXV entry into the host cell, the viral particle can use the rough endoplasmic reticulum to adjust cytoplasmatic machinery, where its replication takes place. Nevertheless, MPXV still needs host cell ribosomes to perform mRNA translation and virion synthesis, allowing genome encapsulation for a posterior cellular exit. It also needs cytoplasmatic proteins of host cells to take apart their endoplasmic reticulum membrane to create their replication system [30].

## 4. Transmission

Poxviruses present two different types of hosts in their cycles: reservoir hosts and zoonotic hosts—the first ones carry the virus and can infect others, whereas the last ones develop the disease. When it comes to MPXV, its reservoir hosts are rodents and squirrels, most specifically species found in the African continent; on the other hand, the zoonotic hosts are monkeys and humans, where the virus can replicate in most mammalian cultured cells [31]. MPXV can also be spread by human-to-human infection. According to the Centers for Disease Control and Prevention (CDC), the human-to-human transmission of MPXV occurs through direct contact with infected skin lesions or prolonged contact of large respiratory droplets with the mouth or nose, although a potential sexual transmission route in the recent 2022/2023 MPXV outbreak indicates amplification of its transmission pattern. Intimate contact is also capable of transmitting MPXV, since sexual relations, hugging, and prolonged face-to-face interaction may promote viral infection as well [14,32].

Due to the recent COVID-19 pandemic, it is important to point out differences in transmissions of the diseases caused by SARS-CoV-2 and MPXV. Firstly, despite the 2022/2023 mpox outbreak, there is already valuable information available about MPXV due to previous research on other viruses from the same family. Secondly, its transmission does not happen in fast contact via respiratory aerosols—as it does with coronavirus: in MPXV it happens via larger droplets of body fluids, which are heavier and thus more difficult to spread through the air and by breathing. Thirdly, the odds of developing the asymptomatic disease are larger in COVID-19 than in mpox disease, which is justified due to mpox presenting visible signs of infection—even in mild infections cases—when compared to the SARS-CoV-2 infection, which does not exclude the possibility of presymptomatic transmission [19,33]. Finally, the presence of fewer strains than RNA viruses is expected, since MPXV is a double-stranded DNA virus [13,27]. However, there is still much that is yet to be discovered on MPXV, such as the possibility of asymptomatic transmission, the frequency of transmission via respiratory secretions, and if it can be transmitted via feces, urine, vaginal fluid, or semen [32]. It is also not clear what environmental circumstances are necessary for MPXV to remain infectious on the surface of objects since it is viable for periods up to 15 days, while other Orthopoxviruses presented longer periods—up to several weeks and months [19]. Although MPXV was detected in all saliva samples collected [34] and it presented higher viral load than oropharyngeal swabs [35], the precise role of MPXV-infected saliva in chains of transmission is yet to be determined, especially when droplets and aerosols are produced during dental clinical procedures [32].

## 5. Oral, Perioral, and Body Symptoms

**Figure 2:**
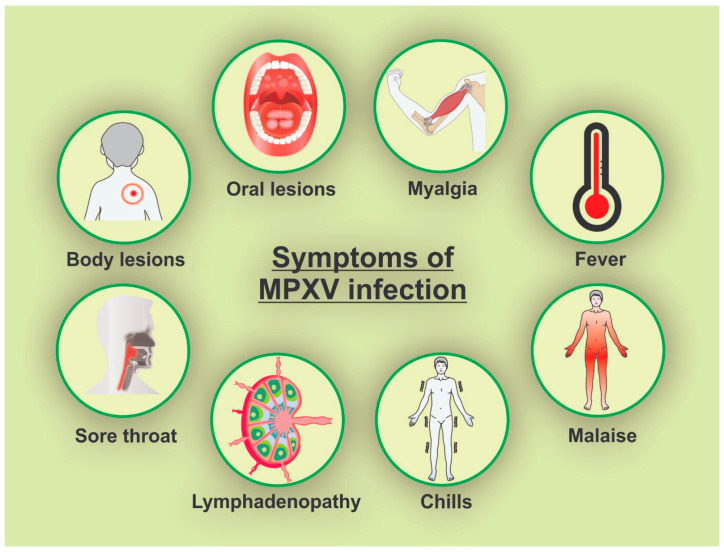
Main MPXV infection symptoms (created by the authors).

Descriptive case series and cohort studies have shown that the presence of oral mucosa lesions associated with mpox infection is one of the first signs of infection. Moreover, oral and perioral lesions could appear in percentages that range from 5% to 70% of the cases [36,37,38,39]. In this context, the lymphadenopathy in the submandibular and cervical areas was described in the majority of the human mpox infections, when associated with inguinal swollen lymph nodes. Compared with smallpox diseases, lymphadenopathy was reported as a characteristic symptom of mpox infection, and odynophagia secondary to mpox-associated ulceration may be present [40,41]. The heterogeneous presentations of the dermatologic, perioral, and oropharyngeal lesions related to MPXV can erroneously be confused with other sexually transmitted diseases [42], thus primary care healthcare workers need improved training in differential diagnosis to test adequately. Compared to other symptoms, oral and perioral lesions can be presented more often than arthralgia, back pain, and axillary lymphadenopathy [43]. Despite lesions caused by mpox infection usually being very heterogeneous, the oral ones seem to follow a well-circumscribed pattern with circular outliners [44]. Other systemic symptoms that can be present are headaches, sore throat, asthenia, myalgia, and proctalgia [33,34,35] (Figure 2).

The identification of intra-oral mpox lesions is key to allowing healthcare workers to recognize the disease stage. During the enanthem stage—which consists of the appearance of a rash or lesions on the mucous membranes inside the body—the lesions first appear in the mouth or tongue, followed by the macular stage, when lesions present a macular appearance. After these first stages, which last one to two days, these lesions tend to progress from macular to raised papular lesions, and from papular to raised vesicular lesions filled with clear fluid—each of these stages also lasts one to two days. For the next five to seven days, the lesions progress into pustules and acquire deep seeded pustular appearance with opaque fluid, round shape, and firm-to-the-touch consistency; then, the lesions develop small depressions to their centers, maintaining this aspect until the end of the five to seven days. By the end of the pustular phase, the lesions begin to crust and to scab over. This process lasts 7–14 days until it is over, and the person is then no longer contagious [15,45].

## 6. Mpox Diagnosis Using Molecular Platforms

The molecular level comprehension of MPXV dynamics including the incubation stage, infectious period stage, and residual DNA shedding stage is pivotal to selecting the best-performing testing strategies for MPXV infection. The sample collection of suspected MPXV-infected subjects offers barriers due to a recent change in transmission mode (i.e., predominantly sexual transmission among MSM) [16]. Based on previous mpox outbreak data, the incubation period for MPXV was predicted to be around two weeks for droplet or noninvasive transmission routes [46]. A shorter incubation period of slightly more than one week was estimated for invasive exposures (mucous membrane) [47]. In the 2022/2023 simultaneous multi-country outbreaks of mpox the incubation stage was estimated to be around 8.5 days based on logarithmic analysis [16,48].

As aforementioned, other diseases with similar clinical characteristics reinforce the need for accurate diagnostic tests. Real-time polymerase chain reaction (RT-PCR) tests are a gold standard platform to perform an accurate and timely detection of MPXV, to support the breaking of chains of transmission. The endorsed specimen for molecular testing confirmation of MPXV is swabs of exudate from skin lesions. The WHO recommended an oropharyngeal swab as specimen type for mpox diagnosis in addition to skin exudate. Rectal swabs, genital swabs, urine, semen, and whole blood are specimens currently recognized for research; and serum and plasma for serology to contribute to diagnosis or research. Despite skin swabs being the standard samples used for MPXV testing, these lesions may be limited and located only in the anogenital area and asymptomatic infections can also occur in mpox patients [49,50]. Moreover, the use of oropharyngeal swabs associated with RT-PCR tests has already detected MPXV, which points out the importance of oral cavities in mpox diagnosis and viral load monitoring [2,51,52]. Until now, mpox diagnostics have been almost performed in symptomatic mpox patients with classical lesions. The RT-PCR testing in multiple samples indicates skin lesions with a sensitivity of 91–100%. It was assumed that the sensitivity of upper respiratory specimens including samples extracted from nasopharyngeal, rectal, and oral swabs, and seminal fluid and saliva reached between 69% and 100% in small-scale studies. RT-PCR analyses were also performed in whole blood, plasma, serum, and urine samples with highly restricted data available to estimate their accuracy properly. Altogether, it is expected that early detection of MPXV infectious, previous to the skin lesions, could be performed by body fluids with distinct sensitivity [16]. Besides, an extended shedding of more than three weeks in several biofluids collected from saliva, skin lesions, oropharyngeal swabs, nasopharyngeal swabs, and urine has been described, however, the clinical relevance and implications for mpox patient care remain unclear [16]. There is hope for the development of a multiplex real-time PCR test that can identify numerous poxviruses at the same time, including MPXV-specific detection [2].

The available diagnostic tests for MPXV detection are performed by real-time quantitative PCR (RT-qPCR). A brief overview of selected attributes of some scalable RT-qPCR assays commercially available to the global population is provided in Table 3 [2]. The target primers were engineered by conserved regions of the central coding and the ITS region. The *E9LNVAR* in the DNA polymerase gene, *B6R* in the envelope protein gene, and *F3L* in the ORF were selected in the central coding region; the *G2R* in a tumor necrosis gene was selected in the ITS region [53].

A multiplex real-time PCR assay for simultaneous detection and differentiation of mpox virus IIa, IIb, and I clades and the B.1 lineage was assembled to detect specific genomic regions (G2R and F3L) of MPXV with unique nucleotide differences between Clades I, IIa, and IIb. Given the rapid appearance of mutations in the 2022/2023 outbreak, permanent evaluation of primers should be performed when new mutations can reduce the performance of diagnostic tests [54]. In low- and middle-income countries with restricted economic resources or places with barriers to access RT-qPCR platforms including thermocycler with electricity supply and temperature control, loop-mediated isothermal amplification (LAMP) tests are an interesting alternative approach. Although it can offer some advantages, LAMP-based test assays still require a moderate laboratory structure. Novel tests based on recombinase polymerase amplification with clustered regularly interspaced short palindromic repeats (CRISPR) associated with lateral flow assays could significantly improve the access of MPXV detection in these areas [16].

**Figure 3:**
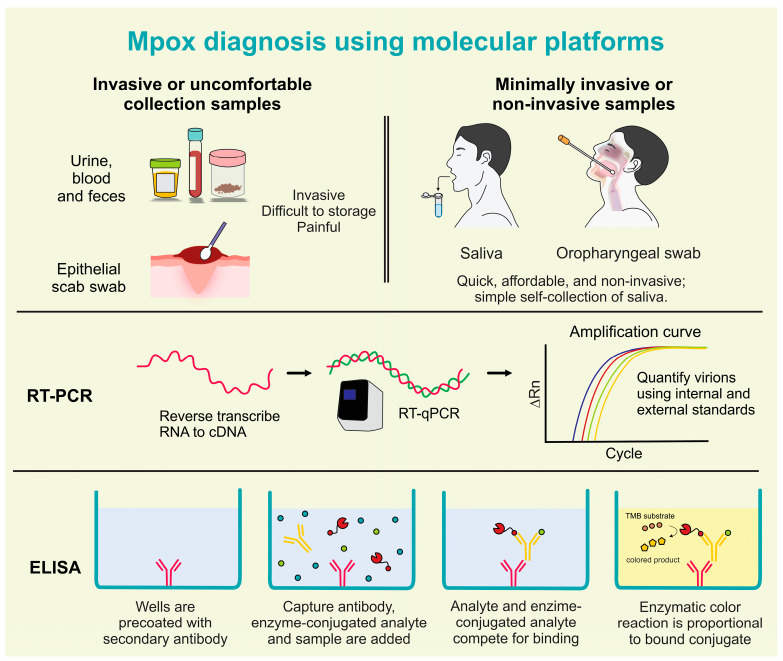
Mpox diagnosis using molecular platforms in invasive, minimally invasive, and non-invasive samples (created by the authors).

Of being executed at BSL2 or preferably in non-controlled places can increase access to mpox tests. Some of these serological tests can inform about the assessment of population immunity, indicating both T cell and B cell immunity responses and also indicates the presence of neutralizing antibodies [55], however, to date, there is a strong limitation of MPXV antigens based on inactivated viral peptides. Another critical limitation is the potential presence of cross-reactivity with other Orthopoxviruses in these serological tests [16]. For IgM and IgG detection in human serum, most serologic techniques currently employ ELISA, where mpox-specific IgM antibodies can be found as early as five days after the beginning of rashes (Figure 3) [2]. In addition, next-generation sequencing technologies such as Ion Torrent PGM, Illumina, and Oxford Nanopore MinION are cutting-edge approaches for mpox diagnosis. These methods enable whole-genome sequencing of MPXV in clinical samples, allowing researchers to get insight into the phylogenomics of circulating mpox strains [2]. Virus isolation and electron microscopy (EM) have also been used since they are critical in virus initial characterization. These procedures are additionally important for virological studies that report on the environmental persistence of the MPXV, as well as viral identification and characterization of poxviruses in experimental settings [51]. All MPXV specimens analyzed should be reported to national health authorities.

## 7. Mpox Salivary Diagnostics and Omics Perspectives on Mpox Research

Before indicating the characteristics of detection of MPXV in saliva, it is important to point out the limitations of the samples currently used for the diagnosis of mpox. In the context of mpox disease, the use of skin lesion exudate presents important barriers to its use in large-scale tests as a result of three main key factors: (i) these lesions can be established in intimate body regions such as the anogenital area, (ii) asymptomatic infections without skin lesions can also occur in mpox patients; and (iii) following the mpox lesions progression in macular, papular, vesicular to pustular stages, the sample collection is different, which may affect the sensitivity and accuracy of current diagnostic tests. In addition, an additional limitation of epithelial scab swab collection is the drastic reduction in lesions in more advanced stages of mpox diseases, which can affect the sensitivity of diagnostic tests. The collection of epithelial scabs using swabs also can present pain at the time of collection, which is another significant disadvantage. The diagnostic of several other infectious diseases occurs using blood samples; however, this collection is invasive, uncomfortable for suspected cases tested, difficult in storage, presents a risk of clotting or even pain at the time of collection, and this collection is not feasible in some healthcare centers [56] These disadvantages drastically limit the large-scale tests that are essential for the control of infectious diseases.

Notwithstanding, saliva samples of infected patients have performed positively in detecting the virus, reinforcing the importance of oral cavity and saliva, which may be a screening or diagnostic specimen as well [35,36,37,45,57,58]. It can be easily self-collected and sample collection does not require expertise [59]. Saliva specimens can be collected in community and household areas where negative ventilation chambers are not available, minimizing the exposure of healthcare workers to the virus and the risk of respiratory cross-infection [60]. This may be relevant to increasing the safety of frontline healthcare workers while analyzing evidence for the contribution of the respiratory route to the transmission of mpox is achieved. Although data analyses indicate little evidence, further large-scale clinical studies need to be performed to assess the potential impact of a respiratory route on mpox transmission [61]. The saliva collection is also simpler and more comfortable than the swab collection, which requires skilled staff with personal protective equipment [57,62]. Overall, saliva has several advantages as a diagnostic fluid due to its ease of collection, low cost, and non-invasive nature.

In a recent study during the 2022/2023 mpox outbreak, MPXV DNA was detected in saliva with 100% sensitivity, frequently with high viral loads compared to other samples [35]. Besides, a viral kinetics study showed that MPXV DNA was detected in saliva 76 days after symptom onset [63]. Another clinical study showed that 88.9% (16 of 18) of mpox-infected patients with skin lesion tests positive also presented the same result in a saliva-based analysis. As expected, in 100% of subjects with a negative test in skin lesions, the results with saliva tests for MPXV DNA detection were similar [64]. The correlation between skin and saliva tests for MPXV DNA detection was also higher in another study achieving 93.5% (29 of 31) of sensitivity to this mpox salivary diagnostics. Moreover, high salivary viral loads of MPXV DNA were detected using RT-qPCR in 85% (35 of 41) of these non-invasive samples. These data were accompanied by other relevant analyses revealing that infectious MPXV virions were rescued from 67% of saliva samples (22 of 33) of mpox patients [65].

There are several potential pathways for MPXV to be present in saliva, including infection of minor and major salivary glands, oral mucosal cells from macules and ulcers, lymph from maxillofacial and cervical lymphadenopathy, and gingival crevicular fluid containing serum-derived proteins [60]. Moreover, MPXV may not be positive in other bodily fluids and swab samples, portraying saliva as plausible and highly sensitive samples, especially when collected three or four days after the onset of symptoms [60]. Although recent advances have occurred in antibody tests for viral diseases, the previous cross-reactivity with antibodies induced by prior smallpox vaccination can disrupt the expected accuracy for MPXV antibody tests [35,40,66]. MPXV antigens and antibodies could be detected using a variety of techniques, including the complement fixation test, hemagglutination inhibition assay, enzyme-linked immunosorbent assay (ELISA), plaque reduction neutralization test, western blot, and electrochemiluminescence (ECL) assay [66]. Real-time PCR and viral isolation are not clearly correlated, especially because viral isolation has only been tested from skin lesion samples, lacking isolation in saliva samples [35]. Still, data on the utility of saliva for MPXV detection is limited, and there is a lack of standard operation procedures for collection methods.

The development of mpox detection using profitable OMICS platforms can be improved with the knowledge of basic molecular mechanisms explaining the pathophysiological routes to mpox-related biomarkers from peripheral tissues to saliva. Until now, there is a scientific gap about the salivary biomarkers of mpox, which limits translation outcomes for developing mpox detection platforms from peripheral tissues to the oral compartment [56].

Salivaomics is a multidisciplinary integrative investigation of saliva components performed by omics platforms [56]. The concept of Salivaomics was first led by a research group at the University of California, Los Angeles (UCLA), which has performed pioneer analysis in the salivary genome, transcriptome, microRNA, proteome, metabolome, and microbiome in translational and clinical studies related to point-of-care salivary devices to detect oral and systemic diseases, including viral infections [67]. With the introduction of high-throughput omics technologies, several reports have been published to comprehensively catalog the salivary proteome in different diseases compared with healthy subjects. In addition, the fact that there is an overlap in protein content between saliva and plasma suggests that saliva could potentially serve as an alternative to blood tests for diagnostic purposes for viral infections [68]. This means that instead of having to draw blood, a simple saliva sample could be used to obtain important diagnostic insights. One explanation for the correlation between salivary and plasmatic proteins is that plasma may flow into saliva; another reason is that plasma and saliva may share important proteins that are required for their physiological roles as bodily fluids [68]. Notwithstanding, viral infectious illnesses may induce changes in salivary protein expression, which can be detected on proteomics-based biomarker discovery [56,68,69]. Research on infectious diseases and chronic diseases based on large-scale multi-omics databases permits a significant improvement in the molecular and genetic features of diseases. It is expected that the integration of multi-omics analysis can promote significant improvement in the performance of diagnostic tests [70]. The integration of salivary omics datasets still presents challenges [67] that can be potentially resolved by artificial intelligence.

It is imperative to note that the identification and quantification of salivary DNA, RNA, protein, and metabolite can provide novel non-invasive biomarkers via omic platforms. The chewing and circadian physiological changes salivary composition, which can hinder the development of saliva-based diagnostic platforms [56]. To date, the framework with computational and informatics strategies does not present specific salivary biomarkers for mpox diseases. However, for transcriptomics, metabolomics, or proteomics analyses, several data of potential marker molecules will be described in these frameworks. A pioneering example is the Salivaomics Knowledge Base (SKB), which was validated at UCLA in an open biomedical initiative [67]. The SKB is a data repository to assist the analysis of miRNA, transcriptomics, metabolomics, proteomics, and also microbiome focused on saliva [67].

Using a mass spectrometry platform, the proteome of MPXV was capable of detecting 152 viral proteins, consistent with ~80% of proteome coverage. In summary, more than 1300 viral peptides were detected and 35 MPXV peptides from 13 viral proteins present higher intensity in mass spectrometry signature, suggesting potential biomarkers in this omics platform with fast and less laborious capacity to detect several viruses simultaneously [71]. A proteomic signature of mpox in blood plasma was obtained in tryptic-digested samples from mpox patients with mild severity of clinical symptoms including skin lesions and respective healthy subjects. It was shown that 56 proteins were differentially expressed in mpox patients compared to healthy subjects, 32 proteins presented higher expression in mpox patients, and the other 24 had lower expression [72]. We point out some proteins with the greatest expression differences comparing mpox patients and healthy subjects, including higher levels of some proteins detected also in saliva as C-reactive protein (CRP) [73], serum amyloid A1 (SAA1) [74], lipopolysaccharide-binding protein (LBP) [75], and leucine-rich alpha-2-glycoprotein 1 (LRG1) [76]. Considering that these proteins were highly expressed in the plasma of mpox patients using a proteomics-based biomarker discovery platform, it suggests that these proteins also detected in saliva are potential salivary biomarkers of mpox disease.

## 8. Measures to Dentists and Frontline Healthcare Workers

The 2022/2023 mpox outbreak is of interest to frontline healthcare workers, including dentists, since they are at risk of being infected, especially considering that the oral cavity is an area closely related to MPXV infection [50,58]. Studies have detected a high viral load of MPXV in saliva, nosocomial transmission of MPXV to healthcare workers has been presented, and it is known that human-to-human transmission can occur via direct contact with bodily fluids, therefore emphasizing the importance of dentists to be careful when assessing patients [39,58,59]. In this context, it is important to emphasize that wearing facemasks and protective goggles is mandatory for frontline healthcare workers, especially because there is the possibility of large saliva droplets coming in contact with the eyes, nose, or mouth of dentists, dental assistants, dental hygienists, and endoscopy suite staffing, which could present a significant risk for infection. In addition, when performing a procedure that generates aerosols in prolonged periods from oral secretions of MPXV-infected patients, the frontline healthcare worker is at risk of being infected by MPXV, reinforcing the aforementioned recommendation of facemask and protective goggles usage [28,40,41]. Nevertheless, all non-urgent dental appointments and endoscopic procedures should be canceled if the patient is infected by MPXV and, if the healthcare professionals get exposed to the virus in the aforementioned scenarios, post-exposure prophylaxis should be performed [50,58]. These infection control measures should be performed without stigma with MSM [77].

Moreover, although human-to-human transmission is described, the mechanisms of how it occurs were not completely elucidated. It could occur via direct contact with contaminated surfaces and with mucocutaneous lesions, leading to viral entry and replication in oropharyngeal and oral mucosa [60]. In this context, A26, A27, D8, and H3 surface proteins of MPXV are critical for viral attachment to the host cell, since they interact with laminin, chondroitin sulfate, and heparan sulfate, which are glycosaminoglycans that are present on the tongue, salivary glands, and oral mucosa [23,25,26,27]. Thus, the oral cavity may play an important role in viral infection and replication, despite these mechanisms still being insipient. In addition, during the incubation stage, the virus circulates to lymph nodes after being absorbed by the mucosa, leading to primary viremia and viral replication on lymphoid organs and distant lymph nodes [57,60,78].

According to the CDC, the National Health Service (NHS), and other relevant dental associations, oral screening could play an important role in the early diagnosis of several infectious diseases, including mpox [2,42,43,45,79]. In addition, thorough patient assessment could lead to easier identification of risk factors, thus allowing the dentist to not only identify these lesions but also provide patient orientation, which could help slow down viral spread [36,37,38,39,41,42,43,80,81,82]. In this context, MPXV was usually detected in the saliva of mpox-infected patients with high viral load, suggesting a potential application in screening or diagnostic platforms for mpox salivary diagnostics.

## 9. Conclusions

The unprecedented 2022/2023 simultaneous multi-country outbreaks of mpox have raised several red flags regarding possible infection routes, especially by frontline healthcare workers who are in direct contact with patients. These professionals, including dentists, can contribute to decrease the spread of the MPXV by understanding novel transmission mechanisms through saliva, respiratory droplets, procedures that produce aerosols and face-to-face contact indicated in the recent 2022/2023 mpox outbreak. More studies are needed to understand viral entrance into host cells and viral replication in the oral cavity, especially approaching laminin, chondroitin, and heparan sulfates, and glycosaminoglycans that can interact with MPXV surface proteins allowing viral attachment and posterior replication in oral cells that express these proteins. The current methods of mpox diagnostics applying molecular methodologies present high accuracy, however, these tests are currently invasive, painful, and rely on the advanced phases of the disease. Notwithstanding, MPXV was detected in the saliva of mpox-infected patients with high viral load, indicating that novel salivary diagnostic platforms can be used in the early diagnosis of mpox. MPXV DNA was detected in saliva with a sensitivity of 85%–100%. In this context, OMICs technologies such as genomics, transcriptomics, and proteomics can be used in the perspective to study the MPXV and unique salivary components in mpox patients. Hence, OMICs technologies could play a central role to prevent future mpox outbreaks by providing detection of novel biomarkers of mpox diseases and aiding in the development of new diagnostic tools applied in saliva samples.

## Figures and Tables

**Table 1:** Expression of genes for proteins receptors in human anatomical entities for Poxvirus based on Bgee platform.

Receptor	Gene Name	Uberon	Anatomical Entity	Expression Score
Laminin (Laminin subunit alpha 1)	*LAMA1*	UBERON:0001830	Minor salivary gland	68.59
Laminin (Laminin subunit gamma 2)	*LAMC2*	UBERON:0001830	Minor salivary gland	79.86
		UBERON:0007371	Superior surface of tongue	59.56
Chondroitin Sulfate (Chondroitin sulfate proteoglycan 4)	*CSPG4*	UBERON:0001830	Minor salivary gland	70.60
		UBERON:0007371	Superior surface of tongue	70.23
Heparan sulfate (Sulfatase 1)	*SULF1*	UBERON:0001830	Minor salivary gland	77.09
		UBERON:0007371	Superior surface of tongue	67.66
		UBERON:0011876	Body of tongue	66.51
Heparan sulfate (Sulfatase 2)	*SULF2*	UBERON:0001830	Minor salivary gland	93.40
		UBERON:0001723	Tongue	88.59
		UBERON:0011876	Body of tongue	88.55
		UBERON:0007371	Superior surface of tongue	87.51

**Table 2:** Expression of proteins receptors for Poxvirus based on the Human Protein Atlas.

Receptor	Gene Name	Anatomical Entity	RNA Expression				Protein
			Consensus nTPM	HPA pTPM	GTEx pTPM	FANTOM5 Scaled Tags Per Million	
Laminin (Laminin subunit alpha 1)	*LAMA1*	Oral mucosa	No data	No data	No data	No data	Not detected
		Salivary gland	1.3	1.3	1.0	4.1	Low
		Tongue	0.0	0.0	No data	0.6	No data
Laminin (Laminin subunit gamma 2)	*LAMC2*	Oral mucosa	No data	No data	No data	No data	Not detected
		Salivary gland	12.9	3.5	12.9	1.3	Low
		Tongue	2.3	2.3	No data	6.3	No data
Chondroitin Sulfate (Chondroitin sulfate proteoglycan 4)	*CSPG4*	Oral mucosa	No data	No data	No data	No data	Low
		Salivary gland	4.7	1.9	4.7	13.0	Medium
		Tongue	10.0	10.0	No data	34.8	No data
Heparan sulfate (Sulfatase 1)	*SULF1*	Oral mucosa	No data	No data	No data	No data	Not detected
		Salivary gland	4.3	2.4	4.3	8.8	Not detected
		Tongue	11.2	11.2	No data	34.1	No data
Heparan sulfate (Sulfatase 2)	*SULF2*	Oral mucosa	No data	No data	No data	No data	Not detected
		Salivary gland	34.0	9.9	34.0	18.3	High
		Tongue	22.5	22.5	No data	22.4	No detected

**Table 3:** Attributes of scalable qPCR assays commercially available for MPXV detection.

Brand Assay	Molecular Target	Processing Workload Capacity	Turnaround Strategy	Features/Analytical Performance
John Hopkins assay	MPXV (E9L), OPXV (B6R)	High-throughput	Variable by cycle threshold	LOD (95%) 100 copies/mL, analytical sensitivity, specificity is adequate and high reproducibility, tested in skin lesion swabs
US CDC assay	OPXV (E9L-NVAR), MPXV (B6R) (Multiplexed)	High-throughput	Variable by cycle threshold	LOD 16 copies/mL, assessed in Routine liquid transport media evaluated for Quantstudio 6
Novaplex MPX assay	MPXV	High-throughput	Variable by cycle threshold	100% sensitivity, evaluated for BioRad CFX 96
Bio-Speedy assay	MPXV (F3L)	High-throughput	Variable by cycle threshold	94% sensitivity, evaluated for BioRad CFX 96
ACON Biotech assay	MPXV (F3L)	High-throughput	Variable by cycle threshold	LOD 250 copies/mL, includes internal control, evaluated for BioRad CFX 96
Altona Diagnostics assay	OPXV	High-throughput	Variable by cycle threshold	Includes internal control, evaluated for BioRad CFX 96
Bioperfectus Technologies	MPXV (F3L)	High-throughput	Variable by cycle threshold	LOD 5 copies/reaction, includes internal control, evaluated for BioRad CFX 96
DaAn Gene	MPXV (F3L)	High-throughput	Variable by cycle threshold	LOD 200 copies/mL, includes internal control, evaluated for BioRad CFX 96
Shanghai ZJ Bio-Tech Co assay	MPXV (F3L)	High-throughput	Variable by cycle threshold	LOD 5000 copies/mL, includes internal control, evaluated for BioRad CFX 96
Perkin Elmer Pkamp Monkeypox Virus RT-PCR RUO kit	MPXV (F3L)	High-throughput	Variable by cycle threshold	LOD 20 copies/reaction, includes internal control, evaluated for BioRad CFX 96
Sansure Biotech Monkeypox Virus Nucleic Acid Diagnostic Kit	MPXV (F3L)	High-throughput	Variable by cycle threshold	LOD 200 copies/mL, includes internal control, evaluated for BioRad CFX 96
Thermo-Fischer Taqman Monkeypox Virus Microbe Detection	MPXV (J1L)	High-throughput	Variable by cycle threshold	LOD < 10 copies/reaction, evaluated for BioRad CFX 96
TIB Mobiol LightMix Modular Orthopox Virus/Monkeypox Virus	OPXV (14kDa), MPXV (J2L/J2R)	High-throughput	Variable by cycle threshold	LOD < 10 copies/reaction, evaluated for BioRad CFX 96
FilmArray Sentinel Panel & FilmArray BioThreat Panel (BioFire)	OPXV	Low-throughput.	60 min	No reagent preparation. One test each per assay.
BD Max	LDT	Low-throughput	180 min	Automated extraction/amplification. Permits to run 24 samples in 3 h.
Roche Omni Cobas 6800/8800 (Roche)	LDT, Cobas MPXV (Multiplexed)	Intermediate-throughput	210 min	Automated extraction/amplification. Permits to run 800 tests in 8 h
Alinity (Abbott Molecular)	LDT, Alinity m MPXV (Multiplexed)	Intermediate-throughput	<60 min	Automated extraction/amplification. Permits A random access, up to 300 tests in 8 h
Panther Fusion Open Access (Hologic)	LDT, can multiplex up to 5 targets	Intermediate-throughput	<140 min	Automated extraction/amplification. Permits random access, up to 800 tests in 8 h.

## Data Availability

Data available upon request.
